# Effects of larval rearing temperature on immature development and West Nile virus vector competence of *Culex tarsalis*

**DOI:** 10.1186/1756-3305-5-199

**Published:** 2012-09-11

**Authors:** Brittany L Dodson, Laura D Kramer, Jason L Rasgon

**Affiliations:** 1 Department of Entomology, Pennsylvania State University, University Park, 16802, PA, USA; 2 Center for Infectious Disease Dynamics, Pennsylvania State University, University Park, 16802, PA, USA; 3 The Huck Institutes of the Life Sciences, Pennsylvania State University, University Park, 16802, PA, USA; 4 Arbovirus Laboratories, Wadsworth Center, New York State Department of Health, Slingerlands, 12159, NY, USA; 5 School of Public Health, State University of New York at Albany, Albany, 12144, NY, USA

**Keywords:** Mosquito, West Nile virus, Global climate change, Transmission, Development

## Abstract

**Background:**

Temperature is known to induce changes in mosquito physiology, development, ecology, and in some species, vector competence for arboviruses. Since colonized mosquitoes are reared under laboratory conditions that can be significantly different from their field counterparts, laboratory vector competence experiments may not accurately reflect natural vector-virus interactions.

**Methods:**

We evaluated the effects of larval rearing temperature on immature development parameters and vector competence of two *Culex tarsalis* strains for West Nile virus (WNV).

**Results:**

Rearing temperature had a significant effect on mosquito developmental parameters, including shorter time to pupation and emergence and smaller female body size as temperature increased. However, infection, dissemination, and transmission rates for WNV at 5, 7, and 14 days post infectious feeding were not consistently affected.

**Conclusions:**

These results suggest that varying constant larval rearing temperature does not significantly affect laboratory estimates of vector competence for WNV in *Culex tarsalis* mosquitoes.

## Background

Temperature is a key factor in vector-borne pathogen transmission. It can significantly affect insect behavior and physiology [1,2] including the extrinsic incubation period of vector-borne pathogens [3]. Studies have measured the effects of temperature on the aquatic immature stages of mosquitoes and other insects [4-6]. Fluctuating temperatures can be experienced by immature mosquitoes residing in containers, irrigation pools and similar ephemeral habitats [7]. In many cases, extremely high temperatures can cause rapid mortality, but moderately high temperatures can also cause thermal wounding at immature stages that affect future adult stages [1]. Low temperatures pose a different set of challenges than high temperatures, invoking complex changes to cell integrity, tissue morphogenesis, reproduction and sex ratio in a variety of insects [8].

*Culex tarsalis* is a primary vector of West Nile virus (WNV), St Louis encephalitis virus (SLEV), and Western equine encephalitis virus (WEEV) in the western United States [9-12]. California field populations have been found persisting in water temperatures as low as 5°C in winter duck ponds or mid-day upper thermal limits of >40°C [13,14]. These conditions are markedly different from those utilized in laboratory settings for colony rearing and vector competence experiments. Adults reared in the laboratory are significantly larger than those collected in the field [15] and lab mosquitoes maintained on sucrose produce and store significantly more glycogen and lipids than their counterparts in the field [16]. Colonization can lead to reduced genotype and phenotype variation [17]. Together, these studies suggest that vector competence experiments with laboratory mosquitoes may not accurately reflect natural vector-virus interactions.

Several studies have shown that the influence of extrinsic factors on immature stages extends to the adult stage, possibly affecting susceptibility to pathogens in a species specific manner. Changes in larval water quality affected *Cx. tarsalis* development parameters including body size, but did not affect vector competence for WEEV or SLEV [18]. Similarly, changes in larval rearing temperature or diet did not alter susceptibility of *Culex spp.* to Rift Valley fever virus (RVFV) or Murray Valley encephalitis virus (MVEV) [19,20]. By contrast, Aedes adults resulting from low rearing temperatures were found to be more susceptible to infection with RVFV, Venezuelan equine encephalitis virus (VEEV) and Chikungunya virus (CHIKV) [5,21]. Others have singly investigated various environmental factors in Aedes mosquitoes such as nutrition deprivation, intra- and interspecific competition, or malathion exposure and found increased susceptibility to La Crosse (LACV), dengue (DENV) and Sindbis (SINV) viruses [4,22,23]. Correlations between small adult Aedes females and increased vector competence for various arboviruses have also been examined [24,25].

Our previous work with *Cx. tarsalis* demonstrated that decreased larval nutrition impacted several development variables including body size, but did not result in consistent changes in adult vector competence for WNV [26]. Immature development, adult size and survivorship have been shown to vary seasonally in field populations of *Cx. tarsalis*[14]. In addition, incidence of WNV, as well as SLEV and WEEV is highly variable seasonably [27-29]. In this study, we investigated the relationship between larval rearing temperatures and vector competence of adult *Cx. tarsalis* for WNV. We also studied impacts of temperature on various development parameters and body size for *Cx. tarsalis.*

## Methods

### Mosquitoes, cells and virus

To control for variation among mosquito genotypes and to account for vector competence changes that possibly occur during the colonization process, two independent *Culex tarsalis* strains were used for experiments. All mosquitoes were reared at the Wadsworth Center Arbovirus Laboratory. Colonies were originally established from field mosquitoes collected in Coachella Valley, CA (“CV”) and Yolo County, CA (“YC”) in 2003 and 2009, respectively, by WK Reisen. Colony larvae, pupae and adult mosquitoes were reared and maintained under the following controlled conditions: 27°C ± 1°C, 16:8 h light:dark diurnal cycle at approximately 45% relative humidity in 30 × 30 × 30 cm cages. Larvae were reared and fed finely ground koi pellets, ground rabbit pellets and bovine liver powder (MP Biomedicals, CA) at a ratio of 1:1:1. Adults were provided goose blood weekly through a membrane feeder for egg development, and 10% sucrose *ad libitum.*

The WNV strain WN02-1956 (GenBank: AY590222) used for all experiments had been isolated from the kidney of an infected American crow in African green monkey kidney (Vero) cells in 2003. Following one round of amplification in *Aedes albopictus* cells (C636), the titer of the virus stock was 5.0E + 09 PFU/ml. Vero cells were used for blood meal virus titrations and screening mosquito samples for WNV. All experiments with infectious virus were completed in the Wadsworth Center, Arbovirus Laboratory Biosafety Level 3 insectary according to established safety protocols.

### Larval treatments

Two replicate experiments were performed each with the CV colony and the YC colony, utilizing three constant temperature treatments at the larval stage (19°C, 25°C and 31°C). For each temperature, five groups of newly hatched larvae (300 per group) were counted into plastic containers (Sterilite, MA) containing 1 L of filtered water. Larval food was added to each container six days per week in the following amounts: 60 mg for 1^st^ and 2^nd^ instar larvae, 90 mg for 3^rd^ instar and 180 mg for 4^th^ instar. Preliminary experiments established that this feeding protocol was optimal for larval development [26].

Pupae for each temperature group were pooled in 50 mL conical tubes filled with filtered water and topped with fine mesh and a sugar cube. Pupal maintenance was continued at the experimental temperatures, on a 16:8 h light:dark diurnal cycle with approximately 45% relative humidity. Newly emerged adults were removed to 4.3 L cartons and held at 27°C until blood feeding. The number of pupae, males, and females were recorded six days per week. Adult mosquito wings were measured from the alular notch to the distal margin, excluding the fringe, using Axiovision software and a Zeiss microscope. Time to pupation, time to adulthood, percent pupation, percent emergence and wing length were compared using chi-squared and t-tests.

### Vector competence

Five-to ten-day old female mosquitoes were starved of sugar for four hours prior to blood feeding. Stock WN02-1956 was added to 5 mL defibrinated bovine blood (Hema-Resource & Supply, Aurora, OR) with 2.5% sucrose solution to average titers of 3.4E + 08 PFU/mL (Table 1). Mosquitoes were fed an infectious blood meal via a Hemotek membrane feeding system (Discovery Workshops, Accrington, UK) for approximately one hour according to the manufacturer’s specifications. After feeding, fully engorged females were separated from the unfed under CO_2_ anesthesia on a FlyPad (Flystuff, San Diego, CA) and placed in 4.3 L cardboard containers. Blood fed adults were provided with 10% sucrose *ad lib* and held for up to 14 days after feeding at 27°C, 16:8 light:dark photoperiod in a growth chamber.

**Table 1:** Final WNV (WN02-1956) blood meal titers in experiments (PFU/mL)

**Temperature**	**Exp 1 (CV)**	**Exp 2 (CV)**	**Exp 3 (YC)**	**Exp 4 (YC)**
19°C	5.0E + 08	4.7E + 08	2.4E + 08	1.4E + 08
25°C	5.8E + 08	4.1E + 08	1.8E + 08	1.3E + 08
31°C	5.8E + 08	5.1E + 08	2.0E + 08	1.4E + 08

*YC* Yolo County, *CV* Coachella Valley.

*In vitro* transmission assays were performed as previously described [26] with modifications at 5, 7 and 14 days post feeding. Female mosquitoes were anesthetized with triethylamine (Sigma, St. Louis, MO), legs were removed and placed in 1 mL mosquito diluent (MD: 20% heat-inactivated fetal bovine serum (FBS) in Dulbecco’s phosphate-buffered saline, 50 ug/mL penicillin/streptomycin, 50 ug/mL gentamicin and 2.5 ug/mL fungizone) per mosquito. For each mosquito, the proboscis was placed in a capillary tube containing 10 uL of a 1:1 solution of 50% sucrose and FBS. After 30 min, the contents were expelled into 0.3 mL MD. One wing per mosquito was removed and retained for measurement for use as an indication of body size. Bodies were placed individually into 1 mL MD. Mosquito body, legs and salivary secretion samples were stored at -70°C until tested for virus. Mosquito bodies and legs were homogenized utilizing a mixer mill at 24 cycles/second and clarified by centrifugation for one minute. Mosquito bodies, legs and salivary secretions were tested for WNV infectious particles via plaque assay on Vero cell culture [30]. Infection was defined as the proportion of mosquitoes with WNV positive bodies. Dissemination and transmission were defined as the proportion of infected mosquitoes with positive legs and salivary secretions, respectively. Proportions were compared using Fisher’s exact test.

## Results

### Mosquito development

To examine the consequences of various larval rearing temperatures on *Cx. tarsalis*, development time, numbers of immature and adult mosquitoes, and adult wing length were evaluated (Table 2). Generally, mosquitoes reared at 19°C took the longest time to pupate and emerge, followed by 25°C and 31°C, (Table 2). Unsurprisingly, temperature affected pupation and emergence rates. In general, larvae reared at 19°C and 25°C were more likely to develop into adults than larvae reared at 31°C. YC mosquitoes used in experiments 3 and 4 were more susceptible to larval and pupal death than CV mosquitoes when reared at 31°C. For all experiments, there was a significant inverse relationship between temperature and wing length, with the largest adults produced at 19°C, and the smallest at 31°C.

**Table 2:** **Effect of rearing temperature on *Cx. tarsalis* development parameters**

**Treatment Temperature**	**Exp 1 (CV)**	**Exp 2 (CV)**	**Exp 3 (YC)**	**Exp 4 (YC)**
	Days to first pupation^1^			
19°C	11.6^a^	12.5^a^	11^a^	11^a^
25°C	7.6^b^	8.8^b^	7.6^b^	7^b^
31°C	7.2^b^	7.8^b^	6.6^c^	6.6^b^
	Days to first emergence^1^			
19°C	15.6^a^	17^a^	17^a^	13.6^a^
25°C	10^b^	10.8^b^	10.2^b^	10^b^
31°C	9.2^b^	9.6^c^	7.6^c^	7.6^c^
	% pupation^2^			
19°C	81^a^	69^b^	75^a^	78^a^
25°C	75^b^	80^a^	69^b^	77^a^
31°C	64^c^	68^b^	44^c^	43^b^
	% pupal emergence^2^			
19°C	83^c^	84^b^	78^b^	85^b^
25°C	92^a^	92^a^	84^a^	89^a^
31°C	87^b^	84^b^	50^c^	68^c^
	% emergence larvae to adult^2^			
19°C	67^a^	58^b^	59^a^	67^a^
25°C	69^a^	74^a^	58^a^	68^a^
31°C	56^b^	57^b^	22^b^	30^b^
	% Adult winglength (mm)^1^			
19°C	3.78^a^	3.87^a^	3.82^a^	3.74^a^
25°C	3.30^b^	3.40^b^	3.37^b^	3.44^b^
31°C	3.08^c^	3.13^c^	-	3.13^c^

^1^ t-test.

^2^ χ2 - test.

Data with different superscripted letters in each development parameter are significantly different within each experiment.

*YC* Yolo County, *CV* Coachella Valley.

### Vector competence

Effects of rearing temperature on *Cx. tarsalis v*ector competence was evaluated by infection, dissemination, and transmission of WNV at 5, 7 and 14 days post feeding (dpf) (Table 3). In experiment 1 (CV mosquitoes), infection and dissemination rates among 19°C treatment group were significantly higher at 7 dpf than the mosquitoes reared at 25°C. In experiment 2 (CV mosquitoes), infection rate of 19°C treatment at 5 dpf was significantly higher than 31°C treatment, and the infection rates at 7 dpf of 19°C and 25°C treatments were both significantly higher than those reared at 31°C. Additionally in experiment 2, 14 dpf transmission rate was significantly lower in the 19°C treatment than those reared at either 25°C or 31°C. In experiment 4 (YC mosquitoes), mosquitoes reared at 25°C had a significantly higher infection rate at 14 dpf than at 19°C. However, there were no consistent statistically significant differences in WNV infection, dissemination or transmission rates at any temperature for either mosquito strain (Table 3).

**Table 3:** **Effect of rearing temperature on adult *Cx. tarsalis* vector competence for WNV (WN02-1956)**

**Treatment Temperature**	**Day post feeding**	**Exp 1 (CV)**	**Exp 2 (CV)**	**Exp 3 (YC)**	**Exp 4 (YC)**
		% Infected			
19°C	5	92(50)	83(41)^a^	100(30)	100(30)
25°C		86(50)	80(50)	97(30)	98(50)
31°C		86(50)	60(40)^b^	-	95(20)
19°C	7	94(50)^a^	81(36)^a^	87(30)	100(35)
25°C		76(50)^b^	82(50)^a^	83(30)	100(38)
31°C		88(50)	44(50)^b^	95(20)	92(24)
19°C	14	92(50)	76(34)	89(38)	88(33)^b^
25°C		86(50)	76(50)	100(13)	100(40)^a^
31°C		82(50)	64(50)	-	-
		% Disseminated^1^			
19°C	5	35	59	63	50
25°C		37	40	76	63
31°C		30	46	-	47
19°C	7	74^a^	90	96	86
25°C		53^b^	80	80	84
31°C		73	73	84	91
19°C	14	98	100	100	100
25°C		88	95	92	98
31°C		95	97	-	-
		% Transmitted^1^			
19°C	5	4	3	10	7
25°C		0	3	10	2
31°C		2	4	-	0
19°C	7	15	7	15	9
25°C		8	22	12	16
31°C		9	27	21	14
19°C	14	61	27^b^	65	72
25°C		51	63^a^	54	70
31°C		56	78^a^	-	-

^1^ Calculated based on number of infected mosquitoes following incubation at 27°C.

Values in parentheses indicate number of mosquitoes tested.

Percentages with different superscripted letters in each infection parameter are significantly different within each experiment.

*YC* Yolo County, *CV* Coachella Valley.

Significance tested by Fisher’s exact test.

## Discussion

Investigating effects of environmental conditions on mosquito life cycle parameters is important considering that laboratory rearing protocols are usually not representative of the temperature, nutrition, and density fluctuations immature larval stages experience in the field. Generally, mosquitoes collected from the field are significantly smaller than those utilized in laboratory experiments, and studies have shown various other differences, including adult nutritional reserves [15,16]. Several studies postulate that climate change may alter infectious disease dynamics, which is influenced by numerous vector and host factors [31,32]. The abundance, distribution, and competence of disease vectors are of particular concern because they are easily influenced by climate and have the potential to impact human health through altered pathogen distribution [31]. Most recently, outbreaks of arboviruses including CHIKV, RVFV, dengue and WNV have resurged or emerged in areas where they were previously undetected, suggesting a further increase in vector-borne disease [32-35].

*Culex tarsalis* is a particularly important species because they are found in a variety of habitats and are competent vectors for SLEV, WEEV and WNV [9,11,12,36]. Seasonal and yearly fluctuations of *Cx. tarsalis* vector competence for WEEV and SLEV have been correlated with changing temperature in Kern County, California, indicating that climate change could facilitate the northward expansion of these viruses [28,29,31]. Our results indicate that larval rearing temperature had significant consequences on development, including shorter development time and smaller female body size as temperature increased. Similarly, other larval environment studies have revealed that temperature influences many *Cx. tarsalis* life history parameters, including decreased adult survivorship, adult body size, reproductive effort and immature development time, which was correlated with increasing temperature [14,37]. Additional larval environment conditions have been examined with a variety of other mosquito species and revealed comparable trends [4,23,38,39].

Many studies in Aedes mosquitoes have found that altering environmental parameters not only influences immature development, but also vector competence for arboviruses [5,22,24,39]. We observed no consistent consequences of rearing temperature on the ability of *Cx. tarsalis* to become infected, disseminate or transmit WNV. Previously, it has been shown that changes in rearing conditions had no effect on the vector competence of *Cx. annulirostris* for MVEV, *Cx. pipiens* for RVFV or *Cx. tarsalis* for either SLEV or WEEV [18-20]. However, it is important to note that in our study, the abundance of adult mosquitoes was significantly affected by rearing temperature. There were significantly fewer adults emerging from the larval group reared at 31°C than at 19°C or 25°C in the majority of replicates, providing fewer mosquitos that are able to be infected and transmit virus. Though rearing temperature has no direct effect on vector competence, it has the potential to affect adult mosquito abundance and thus vectorial capacity, although this phenomenon will be influenced by density dependent regulation.

## Conclusions

Our larval studies, in combination with results from other groups, suggest that varying constant larval rearing temperature has no effect on *Culex* species vector competence. It remains to be seen whether fluctuating rearing temperature may affect this phenotype [40]. In addition, temperature was altered while holding other parameters constant; further studies should be conducted to determine whether changing multiple factors simultaneously will affect WNV vector competence in *Cx. tarsalis*.

## Abbreviations

WNV: West Nile virus; WEEV: Western Equine Encephalitis virus; SLEV: Saint Louis Encephalitis virus; RVFV: Rift Valley Fever virus; MVEV: Murray Valley Encephalitis virus; VEEV: Venezuelan Equine Encephalitis virus; CHIKV: Chikungunya virus; DENV: Dengue virus; SINV: Sindbis virus; LACV: LaCrosse Encephalitis virus; PFU: Plaque forming units; CV: Coachella Valley strain; YC: Yolo County strain; FBS: Fetal bovine serum; MD: Mosquito diluent; dpf: Days post feeding.

## Competing interests

The authors declare that they have no competing interests.

## Authors’ contributions

BLD participated in the study design, carried out laboratory experiments, participated in data analysis and participated in drafting the manuscript. LDK participated in the study design and participated in drafting the manuscript. JLR participated in the study design, participated in data analysis and participated in drafting the manuscript. All authors read and approved the final version of the manuscript.
